# Oral health-related quality of life and dental caries in rheumatoid arthritis patients: a cross-sectional observational study

**DOI:** 10.25122/jml-2022-0081

**Published:** 2022-06

**Authors:** Aida Mehdipour, Maryam Masoumi, Parisa Shajari, Mohammad Aghaali, Hoda Mousavi, Ali Saleh, Miad Ansarian

**Affiliations:** 1 Department of Pediatric Dentistry, Qom Dental Faculty, Qom University of Medical Sciences, Qom, Iran; 2 Cellular and Molecular Research Center, Qom University of Medical Sciences, Qom, Iran; 3 Clinical Research and Development Center, Shahid Beheshti Hospital, Qom University of Medical Sciences, Qom, Iran; 4 Student Research Committee, School of Dentistry, Qom University of Medical Sciences, Qom, Iran; 5 Department of Community Medicine, School of Medicine, Qom University of Medical Sciences, Qom, Iran

**Keywords:** rheumatoid arthritis, oral health, dental caries, oral health-related quality of life, dental decay, DMFT

## Abstract

Rheumatoid arthritis (RA) is a systemic, chronic, and inflammatory joint disease with oral complications. This research aimed to compare the oral health-related quality of life and decayed, missing and filled teeth (DMFT) index in rheumatoid arthritis patients over 18 years with healthy individuals. In this study, 45 rheumatoid arthritis cases were assigned to the experimental group, and 45 healthy individuals were assigned to the control group. After completing biography forms, the participants filled out two questionnaires. These questionnaires included the Oral Health Impact Profile-14 (OHIP-14) and the Oral Health Assessment Index (GOHAI). Next, their teeth were clinically examined to check for caries. Finally, the data were analyzed statistically. RA and control groups were similar in gender, marital status, age, occupation, and level of education. However, a significant difference was observed between the two groups concerning DMFT (P<0.001) and total OHIP-14 score (P<0.001). Moreover, no significant difference was observed between the groups concerning the total GOHAI score (P=0.526). The oral health-related quality of life in rheumatoid arthritis patients was lower than that in the general population, with the rate of dental caries being higher in these patients.

## INTRODUCTION

Rheumatoid arthritis (RA) is an illness with a rate of 0.5 to 1% in the population, which causes chronic synovial inflammation, especially in movable joints; it also malfunctions joints, destroys bones, and degenerates them, with systemic complications [1, 2]. In the course of this disease, interleukin-1, as a pathological factor, causes inflammation and activates the immune response in joints and bones. In addition, interleukin-2 causes joint damage by stimulating synovial cells to produce prostaglandins and collagenase [3]. This disease is treated by suppressing symptoms using a variety of drugs, such as aspirin, non-steroidal anti-inflammatory medicines (NSAIDs), cyclooxygenase-2 (COX2) inhibitors, oral glucocorticoids, tumor necrosis factor (TNF)-neutralizing drugs, as well as immunosuppressive drugs, such as methotrexate, with each of these leading to numerous side effects [4].

Oral manifestations are among the major side effects of these drugs, which appear in the oral mucosa or other manifestations, such as xerostomia, periodontal diseases such as alveolar bone resorption, and tooth loss [5–7]. Moreover, temporomandibular joint (TMJ) involvement has been observed in 1 to 60% of rheumatoid arthritis patients. Sjögren's syndrome may predispose 30% of patients with periodontal diseases and tooth decay by reducing the salivary flow and adhesion of nutrients to the gums and dental surfaces, thereby reducing the salivary buffering property and enamel demineralization remineralization balance [2, 8, 9]. In *Streptococcus mutans* and *Streptococcus subrinus* dental caries, the two bacteria are associated with non-oral infections, such as subacute bacterial endocarditis, atherosclerosis, coronary artery disease, and other systemic conditions [10, 11]. Untreated dental diseases and poor gum health can be risk factors for systemic infections by developing oral infections, especially when using immunosuppressive drugs [12]. Research shows an association between increased caries in RA patients, but given inconsistencies in the findings of previous studies, there is still insufficient evidence in this regard. Given the preventability of dental caries [2, 12–16], investigating the prevalence of dental caries in people with rheumatoid arthritis, preventing their development, and their timely treatment can be effective in reducing the risk of diseases such as endocarditis and rheumatoid arthritis.

Oral Health-Related Quality of Life (OHRQoL) is a particular facet of general health [4]. Previous studies evaluated OHRQoL in rheumatoid arthritis patients, indicating a decrease in OHRQoL compared to healthy individuals [15,17–19]. Problems associated with the disease or medications, including xerostomia, may decrease OHRQoL in rheumatoid arthritis cases [20]. The need for regular oral care in the management of juvenile arthritis has been frequently emphasized; however, poor oral health, high amounts of dental caries, untreated caries, and the high rate of tooth loss indicate difficult oral care in these patients [12]. Accordingly, these findings indicate the effect of specific complications of rheumatoid arthritis on OHRQoL in these patients. Nevertheless, this issue has been a hypothesis until now and has not been approved through clinical examinations [4].

Early and periodic dental examinations for preventing dental caries and TMJ problems as well as preventing a decrease in OHRQoL among rheumatoid arthritis patients are essential. This indicates the need for investigating OHRQoL and decayed, missing and filled teeth (DMFT) index rates in these patients compared to healthy individuals.

## MATERIALS AND METHODS

This cross-sectional descriptive-analytical research was performed in 2021 in Qom city, Iran.

### Study population

The sample size was calculated based on the study of Martinez et al. [2]. In addition, it was performed upon considering the mean index care in the two groups and comparing the mean formula for 45 people in each group.

A total of 90 eligible individuals (76 females and 14 males) were recruited for the study using the convenience random sampling method after obtaining informed consent forms from the participants.

### Inclusion criteria

Control group: (1) healthy individuals over 18 years old and (2) cooperation. RA group: (1) people aged over eighteen years with rheumatoid arthritis who were diagnosed for at least six months by a rheumatologist based on the criteria of ACR/EULAR 2010 for rheumatoid arthritis sorting and (2) cooperation.

### Exclusion criteria

Patients with other rheumatologic, autoimmune, and systemic diseases, inflammatory conditions, active infection, malignancies, or endocrine and oral disorders unrelated to RA.

### Study design and data collection

The statistical population consisted of 45 eligible individuals, including rheumatoid arthritis patients referred to a private rheumatology clinic in the Qom city of Iran. Patients had to be diagnosed for at least 6 months and no more than 25 years by an expert rheumatologist, according to ACR/EULAR 2010 criteria [21]. Patients were recruited from those referred to a private rheumatology clinic. A total of 23 participants were in the active phase, and 22 participants were in the remission phase of the disease. The control group was chosen from a healthy parent sample>18 years old referred to a pediatric hospital for medical examination of their children.

CRP, ESR, and DAS28 (disease activity score 28) tests were used to analyze the activity of rheumatoid arthritis. The illness was considered in the remission mode if DAS28<3.2, and moderate if 3.2≤DAS28≤5.1. In addition, the disease was considered active if DAS28>5.1 [22].

Both RA and control groups were matched in age and gender. Furthermore, the individuals' demographic information was entered into the information form.

### Dental examination

To evaluate teeth condition, the DMFT index was used to determine the health and function of the teeth by examining their surfaces for decay, filling, or missing. To evaluate DMFT, the teeth were examined under standard light with a mouth mirror and a dental probe. Next, the examined surfaces of the anterior teeth of both jaws, including mesial, distal, palatal, or lingual surfaces, were examined; for the posterior teeth of the two jaws, the mentioned surfaces were examined along with the occlusal surface. Before the examination, the teeth were not brushed, and visible food particles were removed with a probe. Caries lesions were diagnosed clinically and by observation. In addition, radiography was not used at any stage of the study. Teeth no. 8 on all four sides were excluded from the study.

### OHRQoL assessment using GOHAI and OHIP 14 questionnaires

OHRQoL was investigated by applying two questionnaires regarding the quality of life concerning oral health.

The domains examined in the GOHAI questionnaire were the three dimensions of physical aspects, psychosocial aspects, as well as pain and discomfort, as explained by Dolan [23]. The total score of each person was calculated by adding Add-GOHAI from the total of the 12 questions, with a score between 12–60 obtained. The lower the total score was, the lower the level of OHRQoL would be. Group Dt GOHAI=0 (Add GOHAI ≤50) had poor quality of life quality, and Dt-GOHAI=1 (Add-GOHAI ≥50) had moderate-to-high quality of life [24].

OHIP 14 questionnaire consisted of 14 items to evaluate seven possibilities of the effect of oral health conditions on an individual's quality of life: physical pain, functional limitation, psychological discomfort, psychological disability, social disability, physical disability, and handicap. The total score for each person was obtained from the 14 questions, ranging from 0–56. The lower the total score was, the higher the OHRQoL would be [4].

### Statistical analysis

After being introduced in the SPSS Statistics 20.0, the data were analyzed using descriptive statistics (frequency, mean, standard deviation) and analytical statistics, including the chi-square test and independent t-test. Moreover, the relationship between variables and quantitative variables was investigated by the Pearson correlation coefficient.

## RESULTS

A total of 90 participants, including 76 women (84.4%) and 14 men (15.6%), participated in the present study.

The mean age of the control and RA groups were 53.9±7.55 and 53.5±7.99 years, respectively (p=0.839). No statistically significant difference was observed between the groups concerning marital status and educational level (P=0.557, P=0.088).

The mean duration of the disease in rheumatoid arthritis patients was 5.82±6.1years, with a minimum of 6 months and a maximum of 25 years.

No significant difference was observed between the genders regarding the total GOHAI score and the total OHIP-14 score (P=0.781, P=0.31). In addition, no significant difference was observed between the level of education with the total GOHAI score and the total OHIP-14 score (P=0.805, P=0.134). Furthermore, no significant correlation was observed between age with the total GOHAI score and the total OHIP-14 score (P=0.128, P=0.523).

In terms of occupation, only the relationship between the participants' occupations and the total GOHAI score was significant. In addition, a significant difference was observed between the employee and retired groups and between the employee group and the housewife group (P=0.003, P=0.01) (Table 1).

**Table 1:** The relationship between occupation and oral health-related quality of life.

Dependent variable	Occupation (I)	Occupation (J)	Mean difference (I-J)	Std. Error	Sig.	95% Confidence interval	
						Lower bound	Upper bound
**Total GOHAI score**	Housewife	Employee	-5.10448	1.59058	.010	-9.2718	-.9372
		Retired	2.56219	1.59058	.378	-1.6051	6.7295
		Self-employed	-.74084	1.21428	.929	-3.9222	2.4406
	Employee	Retired	7.66667	2.15499	.003	2.0206	13.3127
		Self-employed	4.36364	1.89434	.105	-.5995	9.3268
	Retired	Self-employed	-3.30303	1.89434	.308	-8.2662	1.6601

### Evaluation of dental caries

The mean number of decayed teeth (p=0.004) and missing teeth (p=0.026) in the rheumatoid arthritis patients were significantly higher than that of the control. However, in terms of the mean number of teeth filled due to caries, the two groups were not significantly different (P=0.815). In addition, a statistically significant difference was observed between the groups concerning the DMFT index (P<0.001) (Table 2).

**Table 2:** Caries in the two groups based on the DMFT index.

Dental status	Group	Mean	Standard deviation	Significance level
**Decayed**	Control	1.93	1.86	0.004
	RA	3.84	3.90	
**Missed**	Control	5.47	6.31	0.026
	RA	9.09	8.66	
**Filled and restored**	Control	3.71	4.05	0.815
	RA	3.93	4.88	
**DMFT index**	Control	11.11	5.90	<0.001
	RA	16.87	6.76	

### Oral health-related quality of life (OHRQoL) investigation

The total GOHAI score was 32.9±4.8 in the rheumatoid arthritis group and 33.4±2.9 in the control group. In general, the difference between the groups was statistically insignificant (p=0.526). However, in the three domains of pain (being uncomfortable when eating and the use of drugs to relieve pain, teeth, gums, and sensitivity to hot/cold temperatures) (P=0.001) and physical disability (having troubles biting and chewing food, being prevented from speaking, being uncomfortable to swallow) (p=0.004), and psychology (p=0.002), the difference between the groups was statistically significant (Table 3).

**Table 3:** Comparing the oral health-related quality of life using the GOHAI questionnaire in the two groups.

Factor	Group	Number	Mean	Standard deviation	Significance level
**GOHAI-physical**	Control	45	18.98	1.60	0.004
	RA	45	17.00	4.09	
**GOHAI-pain**	Control	45	12.91	2.07	0.001
	RA	45	11.13	2.81	
**GOHAI-psycho**	Control	45	1.53	2.14	0.002
	RA	45	4.76	6.19	
**GOHAI-total**	Control	45	33.42	2.89	0.526
	RA	45	32.89	4.82	

The total OHIP-14 score was 12.82±14.4 in the RA group and 4.5±5.4 in the control group. Accordingly, it was significantly higher in the RA group than in the control group (p=0.001). A significant difference was observed between the groups in the seven domains of functional limitations (having troubles pronouncing and worsened taste) (p<0.001), physical pain (painful aching and feeling uncomfortable to eat) (p=0.001), psychological discomfort (feeling self-conscious and tense) (p=0.007), and physical disability (finding it difficult to relax and embarrassed) (p=0.001), psychological disabilities (p=0.008), social handicaps (p=0.018), and handicap (p=0.005) (Table 4).

**Table 4:** Comparison of oral health-related quality of life using the OHIP14 questionnaire in the two groups.

Factor	Group	Number	Mean	Standard deviation	Significance level
**Functional limitation**	Control	45	0.47	0.87	<0.001
	RA	45	1.62	1.86	
**Physical pain**	Control	45	1.24	1.46	0.001
	RA	45	2.60	2.33	
**Psychological discomfort**	Control	45	0.84	1.76	0.007
	RA	45	2.18	2.73	
**Psychological disability**	Control	45	0.58	0.97	0.008
	RA	45	1.51	2.08	
**Physical disability**	Control	45	0.56	1.10	0.001
	RA	45	2.04	2.70	
**Social handicap**	Control	45	0.58	0.81	0.018
	RA	45	1.51	2.44	
**Handicap**	Control	45	0.24	0.53	0.005
	RA	45	1.36	2.48	
**OHIP-total**	Control	45	4.51	5.38	0.001
	RA	45	12.82	14.45	

## DISCUSSION

Rheumatoid arthritis, a chronic and destructive disease, affects oral health-related quality of life [14]. According to this research, no significant difference was observed between the groups concerning demographic variables. In addition, no significant difference was observed concerning the individuals' age, gender, educational level, total OHIP-14 score, and total GOHAI score. This finding supports the results of Ikebeet al.(2012) [23].

In terms of occupation, only the relationship between the participants' jobs and the total GOHAI score was significant. In addition, there was a difference between the employed group and the retired group, as well as between the employed group and the housewife group. Considering the small sample size in the current investigation and the lack of sufficient evidence and previous studies on the relationship between individuals' occupation and OHRQoL, further research is recommended.

In terms of tooth decay, the difference between the two groups was significant in all variables except the sum of filled teeth. In addition, the sum of decayed and lost teeth and the overall mean of DMFT in rheumatoid arthritis cases were higher than in healthy individuals. This could be for some reasons, including decreased salivary flows, increased salivary acidity, poor oral hygiene, the emergence of microorganisms, osteoarthritis of fingers and hands, and generalized osteoarthritis of the body, leading to the inability to move organs.

Rita Martinez et al. (2019) reported that the level of DMFT in the control group was higher than that in the rheumatoid arthritis group, with no significant difference between the two groups [2]. However, the analysis of the DMFT index showed that the amount of tooth decay in rheumatoid arthritis cases was higher than in healthy individuals, and the number of filled teeth in healthy individuals was higher than in rheumatoid arthritis patients, consistent with the present study. In the study conducted by Medrano et al. (2022), they reported a significant difference in the number of decayed teeth between rheumatoid arthritis patients and the control group, which was consistent with our findings [14]. However, in their study, unlike the present one, there was no significant difference between the groups concerning DMFT and the sum of lost teeth.The reason for this inconsistency could be the acute phase of the disease in all patients in their study as well as the relationship between the increase in the number of lost teeth and chronic rheumatoid arthritis. According to Muhlberg et al. (2017), the DMFT rate in the RA and control groups was 17.61 and 16, respectively. Accordingly, in their study, no significant difference was observed between the groups [15]. In the study by Javier Silvestre-Rangil et al. (2016), DMFT in rheumatoid arthritis patients and the control group was 11.84 and 10.56, respectively, with no significant relationship between the two groups [16].

The results of the studies above are not according to the present ones. Differences between the results of the mentioned studies and those of the present one could be in terms of the population, culture, lifestyle, socio-economic status, and culture-building activities in the field of oral health in different communities.

According to previous studies, rheumatoid arthritis patients suffer from anxiety, depression, and reduced quality of life due to specific conditions caused by underlying diseases and mental factors [15]. In this study, oral health-related quality of life was evaluated by the two questionnaires of OHIP14 and GOHAI. Accordingly, GOHAI is one of the most common indicators for determining health-related quality of life. Statistical results in the current research showed that the difference in the total GOHAI score was insignificant between the RA and control groups, yet in the three areas of pain and physical and psychological aspects, the difference between the two groups was significant.

In rheumatoid arthritis patients, due to the reduced number of teeth, the need for prosthetic treatments, and an increase in the prevalence of TMJ disorders, which affect the quality of their eating, more problems are observed while chewing food. Periodontitis can be one of the reasons for the reduced number of teeth in these patients. In addition, they have an increased need for receiving drugs to relieve their physical pain, mouth pain, and tooth pain [25]. Although no significant difference was observed between the RA and control groups concerning the total GOHAI score, the total score of oral health-related quality of life was lower in the rheumatoid arthritis cases than in healthy individuals. According to Nosratzehi et al. (2019), the total GOHAI score was 37.46±9.3 and 53.21±11.3 in rheumatoid arthritis and control groups, respectively [24]. Moreover, according to Ahmed et al.(2021), the total GOHAI score was 34.6 and 52.8 in RA and control groups, respectively [26]. In both studies, the difference was significant, which showed lower oral health-related quality of life in rheumatoid arthritis cases. In our study, the difference in the total GOHAI score in the RA and control groups, despite the lower total GOHAI score in the RA group, was not significant. This could be due to differences in the course and severity of rheumatoid arthritis in people investigated in previous studies and those in the present study.

In addition, the total OHIP-14 score in our study was significantly higher in rheumatoid arthritis patients than in the control group. The two groups were significantly different in functional limitations, psychological discomfort, physical pain, physical disabilities, social handicaps, psychological disabilities, and handicaps.

The patients' concern about and awareness of oral problems, as well as their dissatisfaction with the appearance of their mouth and teeth, indicate significant effects of oral problems on the psychological state of these people. In reality, OHRQoL has a significant effect on people's well-being and satisfaction with life. However, the social life of this group of patients is more affected by the pain and physical limitations caused by disease than their oral and dental disorders [25]. The study conducted by Ahola et al. on OHRQoL in rheumatoid arthritis patients, for the first time, indicated a significant difference between the groups (rheumatoid arthritis and healthy groups) concerning the total OHIP14 score[19]. Thus, their study was in line with the present one. This is explained by oral discomfort, reducing the quality of life in rheumatoid arthritis cases. Moreover, Rodrigues et al. indicated that the mean score of OHIP14 in rheumatoid arthritis and healthy groups was significantly different [27]. Accordingly, this difference was significant in all domains, with the results somewhat similar to those of the present study.

The findings of Muhlberg et al. were similar to ours regarding the difference in the total OHIP14 score between RA and control groups [15]. According to this study, in the rheumatoid arthritis group, there was a significant increase in the total OHIP14 score compared to that in the general population. In addition, this amount was higher than that in other oral diseases, such as TMD, periodontitis, and gingivitis, confirming that rheumatoid arthritis patients have more unpleasant feelings regarding oral disorders than other people with oral diseases and arthritis. This had a significant effect on reducing the quality of life of these people. Among the reasons for such unpleasant feelings are physical defects and limitations in performing delicate movements due to the disease, which reduce the efficiency and quality of oral health measures, leading to a decrease in their quality of life. The other reason is that rheumatoid arthritis patients suffer from chronic pain, fatigue, and depression, with past research having reported an association between depression and rheumatoid arthritis. These complications are likely to reduce the quality of life in patients [15, 19, 27].

Based on Blaizot et al., there was no significant relationship between demographic factors and GOHAI, in line with our findings. Some studies reported that hand movements are limited and impaired in RA patients. Brushing frequently but improperly results in oral health problems as well as periodontal and dental decay diseases, thereby leading to tooth loss in the long term. This condition can eventually lead to oral self-image disorders in these patients. In addition, a number of other factors, such as limited access to dental care or reduced salivary flows in RA patients, may affect the relationship between the disease and poor oral health-related quality of life [25].

The present study had numerous limitations and problems caused by its coincidence with the COVID-19 pandemic. Some of the problems and limitations included reduced visits of patients, transportation problems, the longer time required for interviewing and examining the participants, and their reluctance to undergo clinical examinations for the risk of contracting COVID-19. Nevertheless, in this study, oral health-related quality of life and caries index of rheumatoid arthritis patients were assessed using two questionnaires, and the findings were then compared with those of healthy individuals, being one of the strengths of this study. Thus, it is recommended that further research should be conducted to quantitatively and qualitatively investigate the bacterial profile of dental plaque and saliva quality in these patients and compare them with those of healthy individuals. Moreover, it is recommended to examine the effect of therapeutic interventions on oral health and oral health-related quality of life in RA patients.

## CONCLUSION

The present study showed that reduced oral health-related quality of life and the dental caries index were greater in rheumatoid arthritis patients than in the general population. Therefore, early and periodic dental examinations can effectively promote oral health and improve the quality of life in rheumatoid arthritis cases.
